# Subsoil-potassium depletion accounts for the nutrient budget in high-potassium agricultural soils

**DOI:** 10.1038/s41598-021-90297-1

**Published:** 2021-06-02

**Authors:** Adrian A. Correndo, Gerardo Rubio, Fernando O. García, Ignacio A. Ciampitti

**Affiliations:** 1 Department of Agronomy, Kansas State University, 3031 Throckmorton Plant Sciences Center, Manhattan, KS 66506 USA; 2 Soil Fertility Laboratory, School of Agriculture and INBA CONICET-, University of Buenos Aires, Av. San Martin 4453, C1417DSE Buenos Aires, Argentina; 3 Facultad de Ciencias Agrarias, Universidad Nacional de Mar del Plata, Ruta 226 km 73.5, B7620 Balcarce, Buenos Aires Argentina

**Keywords:** Plant sciences, Element cycles, Geochemistry

## Abstract

Continuous potassium (K) removal without replenishment is progressively mining Argentinean soils. Our goals were to evaluate the sensitivity of soil-K to K budgets, quantify soil-K changes over time along the soil profile, and identify soil variables that regulate soil-K depletion. Four on-farm trials under two crop rotations including maize, wheat and soybean were evaluated. Three treatments were compared: (1) control (no fertilizer applied); (2) application of nitrogen, phosphorus, and sulfur fertilizers -NPS-; and (3) pristine condition. After nine years, crops removed from 258 to 556 kg K ha^−1^. Only two sites showed a decline in the exchangeable-K levels at 0–20 cm but unrelated to K budget. Topsoil exchangeable-K levels under agriculture resulted 48% lower than their pristine conditions, although still above response levels. Both soil exchangeable-K and slowly-exchangeable K vertical distribution patterns (0–100 cm) displayed substantial depletion relative to pristine conditions, mainly concentrated at subsoil (20–100 cm), with 55–83% for exchangeable-K, and 74–95% for slowly-exchangeable-K. Higher pristine levels of exchangeable-K and slowly-exchangeable-K and lower clay and silt contents resulted in higher soil-K depletion. Soil K management guidelines should consider both topsoil and subsoil nutrient status and variables related to soil K buffer capacity.

## Introduction

Potassium (K) is an essential macronutrient required by crops, which exhibit a broad range of K requirements (40–300 kg ha^−1^ year^−1^) and K removal from the harvested product^1^. Wheat (*Triticum aestivum* L.) and maize (*Zea mays* L.) crops export around 3 kg K Mg^−1^, whereas soybean (*Glycine max* L.) exports 16 kg K Mg^−1^^2–4^. The main source of plant K is the soil, where K is distributed in four different pools: (1) soil solution K, (2) exchangeable K (NH_4_-OAc-K), (3) slowly exchangeable K (NaBPh_4_-K), and (4) K-bearing primary minerals^5–7^. The soil solution-K and NH4-OAc-K fractions are the most readily available K source to plants. Thus, fertilizer recommendations are mostly based on these fractions^8^. The NaBPh4-K pool includes less available K fractions albeit has received more attention for diagnosing K fertilization needs during the last decades^9–11^. Finally, the mineral-K is a recalcitrant soil K fraction, usually not considered for K diagnosing purposes.

Potassium can be acquired from all soil layers explored by plant roots, depending on the crop species and on vertical distribution of plant available K^12^. Although root mass is concentrated at topsoil, maize root mass was reported as poorly correlated with root activity, which can remain high until 60 cm depth^13^. Spring cereals might take up to 80% of the K from the 0–25 cm layer^14^, while soybeans may take about to 60% of the K from the 0–30 cm layer^15^.

Studies at a global scale indicate that the stratification towards the topsoil is less marked for K than for other major nutrients such as phosphorus and nitrogen^16^. Thus, only a portion of the soil K available for plants is captured if soil samples are collected from the topsoil (i.e. 0–20 cm depth)^17^. This underestimation is reduced in those soils with a high topsoil K stratification^14^. Stratification normally occurs when K fertilizer is applied in the topsoil^18^, as well as when roots uptake K from deeper soil layers and residues are deposited in the topsoil^16^. This process is enhanced in fertilized and high-yielding systems, involving a high deposition of plant residues such as in no-tillage systems^19^.

Most agricultural soils of the Argentine Pampas are characterized by high native soil K linked to the clay mineralogy (predominantly illite) of the parental material^20^. Positive yield responses to K fertilization have been rarely observed in the region so far; thus, fertilizer K applications have been negligible during the Argentinean agricultural era, which started around 100 years ago. However, continuous K removal via harvest is gradually depleting soil K reservoir^21^. Under comparable negative K budgets in Pampean soils, greater NH_4_-OAc reductions (46% vs. 8%) at the topsoil were found in soils with high- (900 mg kg^−1^) relative to low-initial (600 mg kg^−1^) NH_4_-OAc-K levels^22^. However, most of Pampean soils are still above the NH_4_-OAc-K critical level (about 130–170 mg K kg^−1^)^21^.

The aim of this study was to quantify the effect of contrasting K removal on soil K fractions along the soil profile. We hypothesize that: (1) in high native K soils, the topsoil (0–20 cm) NH_4_-OAc test is not sensitive to identify short-medium term K depletion trends; (2) vertical distribution patterns of both NH_4_-OAc-K and NaBPh_4_-K are affected by the agricultural footprint, with a significant depletion expected to occur beyond the topsoil (0–20 cm); and (3) soil K deviates from the Pristine conditions depending on soil factors related to K buffer capacity such as soil texture and indigenous richness of soil K, with more depletion expected to occur for both K fractions with coarser textures and high pristine soil K levels.

## Results

### Cumulative K budget

**Figure 1:**
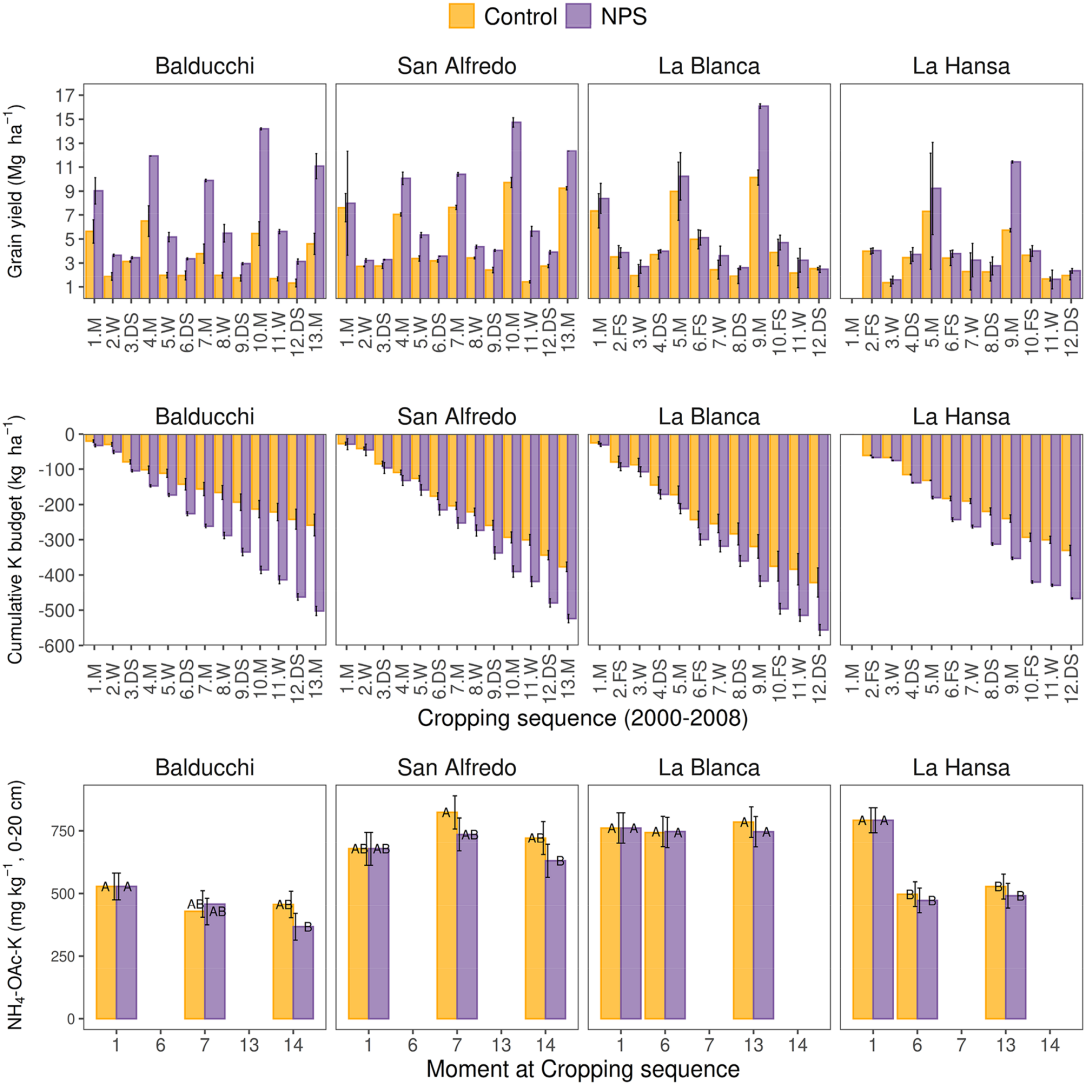
Grain yield, cumulative K removal by grain (K budget), and NH_4_-OAc-K at 0–20 cm for check treatment with no fertilizers added (Control) and fertilized plots with N, P and S (NPS) for each experimental site under two different cropping sequences (Table 1) from 2000 to 2009. Each observation is the average of three replicates. Error bars represent the standard deviation for grain yields, and standard errors from their respective analyses of variance for cumulative K budget and topsoil NH_4_-OAc-K (Tukey’s HSD, *p* < 0.05). Within each site, different letters indicate significant differences between NH_4_ -OAc-K levels at 0–20 cm for treatment-moment at the sequence combinations (Tukey's HSD, *p* < 0.05). *M* maize, *W* wheat, *DS* double cropped soybean, *FS* full season soybean.

Cumulative K budget reflects continuous K removal, without K fertilization. The high-yielding system, NPS treatment (Fig. 1), significantly increased K removal, with an average annual K removal ranging from − 29 to − 62 kg K ha^−1^ year^−1^. After more than 10 harvests, the cumulative K budget for the Control varied from − 258 to − 421 kg K ha^−1^ and for the NPS ranged from − 467 to − 556 kg K ha^−1^ (Fig. 1). San Alfredo, La Blanca and La Hansa sites presented comparable K removal on Control plots (− 330 to − 421 kg K ha^−1^), whereas Balducchi exhibited lower K removal (− 258 kg K ha^−1^). Cumulative removal at the NPS resulted similar across sites (− 467 to − 556 kg K ha^−1^). Differences in K removal are largely explained by final yields, with lower yield for the Control in Balducchi but with similar yield for the NPS relative to the other sites (average 1.9 vs. 3.2 Mg ha^−1^, with larger yield gap for Control and NPS, respectively).

### Topsoil NH_4_-OAc-K changes

The evolution of NH_4_-Oac-K in the topsoil (0–20 cm) was site-specific (Fig. 1). The sites with longest agricultural history at the beginning of the experiment (Balducchi and La Hansa), presented significant NH_4_-OAc-K reductions from 2000 to 2009. The opposite occurred in those sites with shorter farming history at the beginning of the experiment (< 10 year: San Alfredo and La Blanca), with no differences between initial and final NH4-OAc-K levels. Surprisingly, no differences were detected between Control and NPS in any case (Fig. 1, Table 2). Thus, the lack of change or negligible reductions in topsoil NH_4_-OAc-K over cropping seasons did not correspond with the increasingly negative K budgets.

### Vertical distribution patterns of soil KNH_4_-OAcK

**Figure 2:**
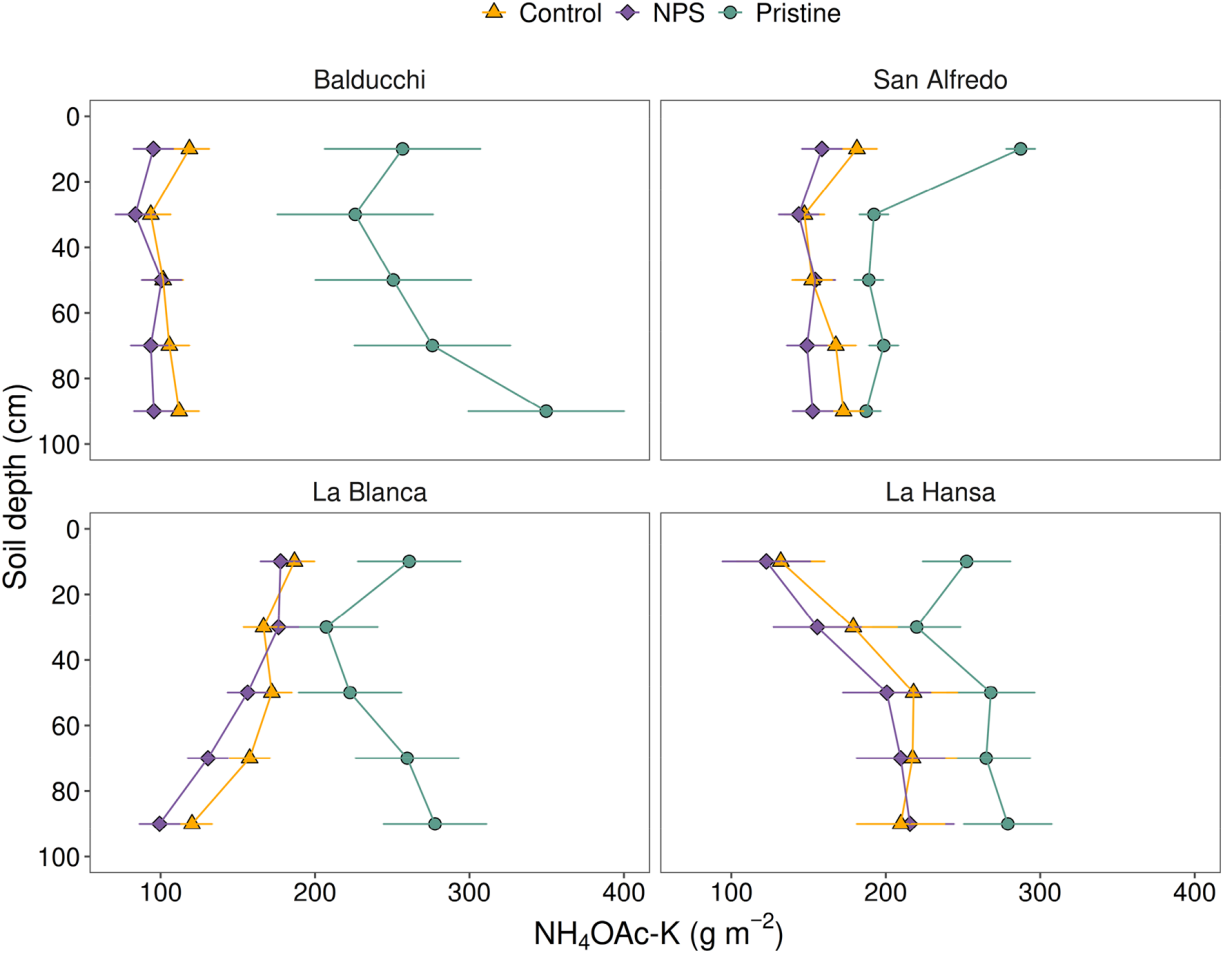
Soil profiles of NH_4_-OAc-K (g m^−2^) under three different conditions: pristine soils (green circles), under grain cropping from 2000 to 2009 with no fertilizers added (Control, orange triangles), and under grain cropping from 2000 to 2009 with N, P, plus S fertilization (NPS, purple diamonds). Overlapping error bars indicate absence of significant differences between scenarios by soil depths combinations (Tukey’s HSD, *p* < 0.05).

The NH_4_-OAcK fraction was predominantly located (range 72–86%) in the subsoil (20–100 cm) with significant differences between sites and treatments (Table 2). Under the Pristine conditions, the distribution in depth showed notorious differences among the four sites (green circles; Fig. 2). The most contrasting soils were Balducchi and San Alfredo, portraying greater NH_4_-OAc-K towards the subsoil and topsoil, respectively. La Blanca and La Hansa sites presented a fairly similar Pristine NH_4_-OAc-K pattern, following a sinuous trajectory with minimum levels at 20–40 cm, and rather comparable values at shallower and deeper layers. Interestingly, observed differences among soils were not reflected in the average NH_4_-OAc-K stocks (0–100 cm), that were rather equivalent (1359, 1055, 1228, and 1284 g m^−2^ for Balducchi, San Alfredo, La Blanca and La Hansa, respectively).

A remarkable effect of agriculture was reflected in a considerable decrease in the NH_4_-OAc-K levels for the Control and NPS relative to their Pristine condition (Table 2; Fig. 2) although its vertical distribution varied depending on the location (Fig. 2). At all locations, significant interactions between treatment and soil depth were documented (Supplementary material 1, Appendix A). Balducchi and La Blanca (Hapludolls), showed the higher NH_4_-OAc-K depletion rates (averaging 63 and 37%; respectively), which was exacerbated towards the subsoil (60–100 cm). Contrastingly, in San Alfredo and La Hansa (Argiudolls), NH_4_-OAc-K reductions due to agriculture were lower (average 25 and 27%, respectively) and occurred mainly towards the topsoil (Table 2). Differences in NH_4_-OAc-K between the Control and NPS showed a lower magnitude than relative for the difference of those treatments to the Pristine due to differences in time-spans (Cropping history, Table 1).

**Table 1:** Soil taxonomy, cropping history, and soil properties (0–20 cm and subsoil layers) at the onset of the experimentation term (September 2000) at four sites of the CREA Southern Santa Fe Crop Nutrition Network.

Cropping sequence	Depth (cm)	pH	SOC (g kg^−1^)	Ca (mg kg^−1^)	Mg (mg kg^−1^)	K (mg kg^−1^)	Clay (%)	Silt (%)	Sand (%)	BD (g cm^−3^)
M-W/DS	**Balducchi (Typic Hapludolls; + 60 year)**									
	0–20	6.3	13.5	1380	252	528	16	43.1	40.9	1.3
	20–40	–	–	–	–	–	23.5	39.1	37.4	1.29
	40–60	–	–	–	–	–	21.7	39.5	38.8	1.3
	60–80	–	–	–	–	–	18.5	41.8	39.7	1.31
	80–100	–	–	–	–	–	16.7	42.9	40.4	1.31
	**San Alfredo (Typic Argiudolls; 8 year)**									
	0–20	6.0	19.8	2200	252	678	24.1	63.3	12.6	1.26
	20–40	–	–	–	–	–	30.9	55.1	14	1.29
	40–60	–	–	–	–	–	33.3	52.6	14.2	1.3
	60–80	–	–	–	–	–	31.7	52.8	15.5	1.31
	80–100	–	–	–	–	–	28	54.9	17.1	1.31
M-FS-W/DS	**La Blanca (Typic Hapludolls; + 6 year)**									
	0–20	6.6	13.3	1440	240	760	17.4	55.7	26.9	1.19
	20–40	–	–	–	–	–	17.9	54	28.1	1.21
	40–60	–	–	–	–	–	16.1	53.9	29.9	1.23
	60–80	–	–	–	–	–	15.7	53.9	30.4	1.24
	80–100	–	–	–	–	–	11.9	54	34.1	1.24
	**La Hansa (Aquic Argiudolls; + 20 year)**									
	0–20	5.5	12.2	1520	192	792	24.8	72.3	2.9	1.25
	20–40	–	–	–	–	–	36.5	59.9	3.7	1.28
	40–60	–	–	–	–	–	46.7	50.3	3	1.3
	60–80	–	–	–	–	–	47.9	49.3	2.8	1.3
	80–100	–	–	–	–	–	38.7	57.8	3.5	1.3

Two sites were under maize–wheat/double-cropped soybean (M-W/DS) cropping sequence, and two under maize–full season soybean–wheat/double-cropped soybean (M-FS-W/DS). Each determination is the average of three replicates. SOC: soil organic carbon; BD: soil bulk density.

### NaBPh_4_-K

**Figure 3:**
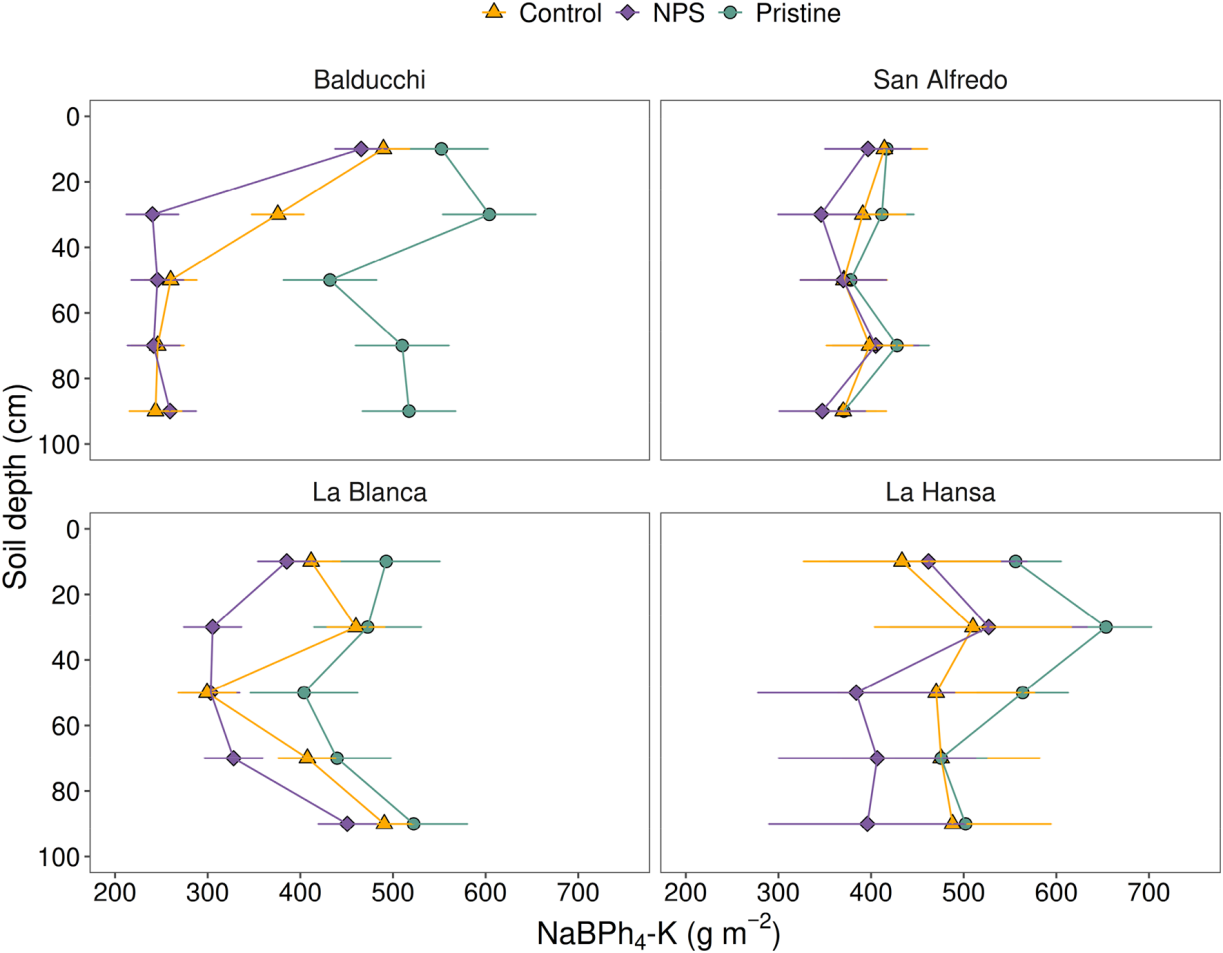
Soil profiles of NaBPh_4_-K (g m^−2^) under three different scenarios: pristine soils (green circles), under grain cropping from 2000 to 2009 with no fertilizers added (Control, orange triangles), and under grain cropping from 2000 to 2009 with N, P, plus S fertilization (NPS, purple diamonds). Overlapping error bars indicate absence of significant differences between scenarios by soil depths combinations (Tukey’s HSD, *p* < 0.05).

Similarly to NH_4_-OAc-K, the slowly-exchangeable fraction (NaBPh_4_-K) was found predominantly in the subsoil (20–100 cm) (range 78–81%), without large differences between sites or treatments (Table 2). The NaBPh_4_-K contents were 2.1 times in average (range 1.2×–6.2×) larger than the NH_4_-OAc-K. Treatment by soil depth interaction was significant (p < 0.05) at Balducchi and La Blanca, while main effects of treatment (p = 0.05) and soil depth (p < 0.05) were significant at La Hansa, and only soil depth resulted in a marginally significant effect (p = 0.11) at San Alfredo (Fig. 3) (Supplementary material 1, Appendix A). Balducchi showed the larger impact of agriculture on their NaBPh_4_-K levels (average 41% depletion as compared to the Pristine scenario), whereas the other sites varied in a rather small range (5–17%).

**Table 2:** Soil-profile K stock (g m^−2^, 0–100 cm depth) for NH4-OAc-K and NaBPh4-K under different agricultural conditions (Pristine, Control and NPS) and its distribution between topsoil (0–20 cm) and subsoil (20–100 cm) layers.

SITE	CONDITION	NH_4_-OAc-K (g m^−2^)			NaBPh_4_-K (g m^−2^)		
		Soil depth (cm)					
		0–100*	0–20^†^	20–100^†^	0–100*	0–20^†^	20–100^†^
Balducchi	Pristine	1359A	257bc	1102a	2615A	552d	2063a
	Control	532B	119c	413b	1615B	490d	1125b
	NPS	469B	95c	374b	1453B	466d	987c
San Alfredo	Pristine	1055A	287c	767a	2005A	417c	1588a
	Control	821B	181d	639b	1945A	414c	1531ab
	NPS	758B	159d	599b	1865A	397c	1468b
La Blanca	Pristine	1228A	261d	968a	2331A	493d	1838a
	Control	803B	187d	616b	2069AB	412d	1658b
	NPS	740B	178d	563c	1772B	385d	1387c
La Hansa	Pristine	1284A	252c	1032a	2751A	556c	2195a
	Control	956B	132c	824b	2378AB	433c	1944ab
	NPS	904B	123c	782b	2176B	462c	1714b

*Within the same site, different capital letters indicate significant differences (Tukey’s HSD, p < 0.05) among scenarios for the soil profile (0–100 cm). ^†^Within the same site, different lowercase letters indicate significant differences (Tukey’s HSD, p < 0.05) at the interaction level (agricultural condition by soil depth).

### Soil K depletion drivers

**Figure 4:**
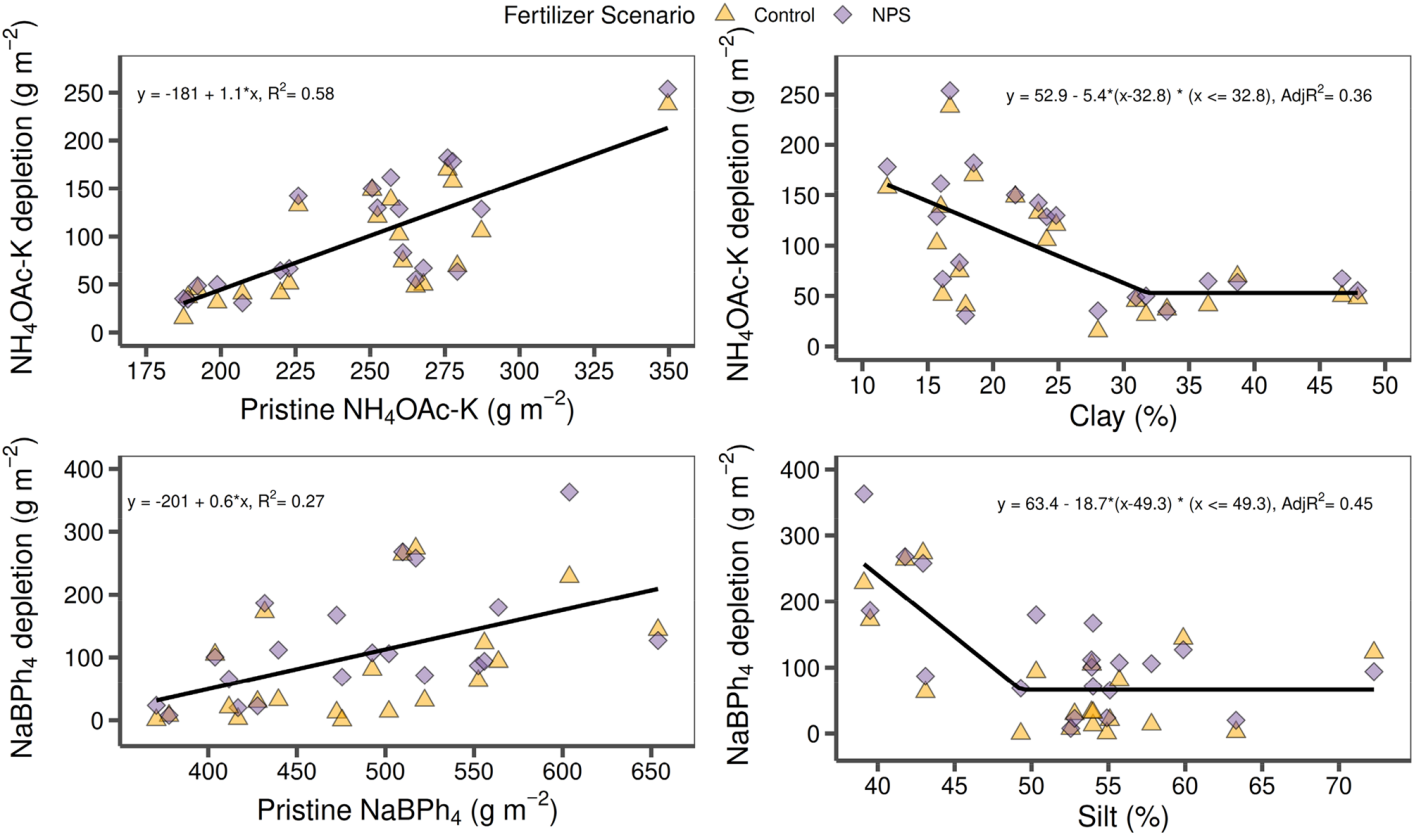
Depletion of NH_4_-OAc-K (upper-panel) and NaBPh4-K (lower-panel) in the agricultural (Control, NPS) with respect to pristine soils as explained by the corresponding pristine-K levels (left) and soil texture related variables, clay and silt content (right). Data points are pooled from the four sites and five soil sampling depths.

The NH_4_-OAc-K depletion patterns followed a close association with the Pristine- NH_4_-OAc-K and clay content of each soil layer (Fig. 4). For the Pristine- NH_4_-OAc-K, the greater the native K values, the larger was the documented depletion K rate. On the other hand, increases in soil clay (%) levels were associated with lower depletion NH_4_-OAc-K rate until reaching a threshold about 33% of clay, remaining at a minimum level for NH_4_-OAc-K depletion with high clay content. Conversely, for NaBPh_4_-K depletion, the most relevant factor resulted the silt content (%), showing a depletion trend until reaching a threshold of 50% of silt, above of which the NaBPh_4_-K depletion becomes minimum. A relatively weak but positive association to Pristine-NaBPh_4_-K content was also observed, however; it resulted less relevant than for the NH_4_-OAc-K depletion.

## Discussion

Agricultural activities without K replenishment led to substantial negative K budgets that were not translated into consistent changes in the topsoil NH_4_-OAc-K fraction (Fig. 1). These results support our first hypothesis, which stated that NH_4_-OAc-K is not highly sensitive at identifying K depletion in these high native K soils. In fact, after nine years of cropping with negative K budgets, only two out of the four sites showed changes in this topsoil K fraction. These two locations (Balducchi and La Hansa) are both characterized by presenting the longest continuous cropping-history, which may have led to greater reductions not only in terms of measured soil K fractions but also related to total soil K. Unfortunately, no historical yield data was available to further explore hypotheses related to the effect of cropping history. Nonetheless, these results would indicate that continuous K removal after nine years of farming has come from extra K-pools (e.g., non-exchangeable-K, root K uplift) rather than the NH_4_-OAc-K at top-soil.

In line with recent surveys performed in the Pampean Region^21^, our four soils remained high in K fertility along the experimental period, as reflected by topsoil NH_4_-OAc-K values above the critical level. Considering an average annual K removal of 62 kg K ha^−1^ (average yields for the studied crop rotation), a soil should lose about 15 mg kg^−1^ of soil K a year. If all the K budget is reflected on the NH4-OAc-K at topsoil, the critical range could be reached in between 6 (Balducchi) to 224 years (La Blanca). Nonetheless, observed variability is in line with Simonsson et al.^23^, who reported that, besides the K budget, the soil K depletion results from the interaction of multiple factors such as cropping history, soil texture and mineralogy and potential subsoil K supply. Additionally, our data reveals that the subsoil NH_4_OAc-K levels are decreasing but still high.

Regarding our second hypothesis, we found evidence to support that K depletion occurs mainly beyond the topsoil. Both soil exchangeable and slowly-exchangeable K vertical distribution patterns displayed large depletion relative to the Pristine scenario in the whole soil profile (0–100 cm). However, depletion of both fractions was more pronounced at subsoil (20–100 cm) (55–83% for NH_4_-OAc-K and 74–95% for NaBPh_4_-K) rather than in the usually tested 0–20 cm soil layer. It is known that plants can remobilize K from the subsoil towards shallow layers^16,24,25^, and this effect would be exacerbated under minimum or no-tillage management where the stover may release significant amounts of soluble K that later has low mobility into the soil matrix^19,26^. Thus, our results highlight the relevance of subsoil on the K supply for crops^27^ and thus, of building awareness about soil K profile concentrations^28^. For practical reasons, most of soil K recommendation systems are based on topsoil data without considering the subsoil—e.g*.* 0–15 cm in the US Corn Belt, where also Mollisols is the dominant soil order^29^. However, our results are consistent with a K recommendation system based on the combined NH_4_-OAc information from both the topsoil and the subsoil such as former system in Iowa^29^ and recommendations in India^30^.

Lastly, obtained results fully support our third hypothesis, since the two main factors affecting K depletion rates were the pristine K levels and the soil texture (reflected as clay and silt content). In the same long-term experiment, Sucunza et al.^31^ reported a similar tendency for phosphorus: the higher the pristine nutrient levels the higher the nutrient depletion rates. In terms of soil texture, its influence on NH_4_-OAc-K critical levels is well documented^9,32,33^, as well as soil K buffer capacity is affected by clay content and mineralogy^34–37^. Particularly in Pampean soils, with *loess* as the main parental material, the silt fraction also contributes to the cation exchange capacity with clay minerals rich in K such as illite^38^, supporting our finding of reduced NaBPh_4_-K depletion with increased silt content. Thus, an increased soil K buffer capacity could mask the effects of continuous K removal. In other soils, negative K budgets were reflected not only in NH_4_-OAc-K but also in changes in more stable and recalcitrant soil K fractions^9,11,24,37,39^. Finally, our results also indicate that the root K uplift process was accentuated at locations without fine-textured horizons (Balducchi and La Blanca). Thus, soil layers with greater clay content involve not only a high buffer capacity but may also denote a mechanical impedance to root growth^40^, ultimately affecting K uptake and removal^41^.

This research offers an indication that topsoil NH_4_-OAc-K interacts with subsoil K and other soil K pools as a K source for satisfying plant nutrient demand. Yet, it is worth acknowledging the need of more comprehensive studies on soil-K depletion trends in response to different crop management practices for the Pampean Mollisols.

## Conclusions

The high indigenous-K fertility of Pampean Mollisols is progressively being depleted due to the prolonged and intensive agricultural use without K replenishment, thereby rendering them to express responsiveness to K in a near future. Continuous K removal by crop harvest without K replenishment led to soil K depletion primarily concentrated in the subsoil. In such sense, the single use of topsoil NH_4_-OAc-K in high native K soils could result in an incomplete assessment of soil K fertility diagnosis. Instead, both topsoil and subsoil K analyses could be complemented to develop more precise guidelines on medium-to-long-term K fertilizer needs. Also, as soil K depletion increased with indigenous K level and decreased with finer soil texture, including them as key metadata will contribute for an improved development of soil K guidelines.

## Methods

### Experimental sites

The Pampas region is located in the East-Central region of Argentina. The climate is temperate and annual mean temperature varies from 15 to 25 °C. The rainfall pattern is humid to the East and semiarid to the West. Mollisols, Entisols, and Alfisols are the predominant soil orders in the Region^42^. Agricultural activities are mainly concentrated in the Mollisols. The main crops are maize and soybean during the spring–summer and wheat during the winter-spring seasons. Maize and wheat are usually fertilized with nitrogen (N), phosphorus (P), and sulfur (S) but at rates below nutrient removal, resulting in negative budgets^43^.

In 2000, a long-term fertilization network was established at farms from the southern Santa Fe region of *Consorcios Rurales de Experimentación Agrícola* (CREA), located in the Central Pampas of Argentina^31,44,45^. Four sites from the network, differing in soil properties and management history, were selected for this study (Table 1). Two sites were under maize–double-cropped wheat/soybean (M-W/S) rotation—Balducchi (34° 9′ 26.0″ S, 61° 36′ 33.8″ W) and San Alfredo (33° 53′ 14.1″ S, 61° 27′ 30.5″ W), and two sites were under maize-full-season soybean-double-cropped wheat/soybean (M-S-W/S) rotation-La Blanca (33° 29′ 57.2″ S, 62° 37′ 55.3″ W) and La Hansa (32° 23′ 04.2″ S, 61° 11′ 58.8″ W).

### Design and measurements

The experimental layout was a randomized complete block design with three replicates. Plot size was 25–30 m wide and 65–70 m long. Two contrasting fertilizer treatments were evaluated: (1) control, without fertilization, (2) the recommended nutrient management, including the three most deficient nutrients in the region—N, P, and S (NPS). Additionally, adjacent soils to experimental plots with no antecedents of agriculture were evaluated as pristine soil conditions (Pristine). For the NPS treatment, N and S were applied at rates based on crop demand, ranged from 90 to 175 kg N year^−1^ and from 17 to 25 kg S year^−1^, P rates were decided each season before planting according to the attainable yield and P removal, averaging 37 kg P year^−1^. Nitrogen fertilizer was not added to soybean. All nutrients were applied before or at planting using solid fertilizer blends. The source of N was urea (46% N), for P was mono-ammonium phosphate (23% P), and for S was calcium sulfate (19% S).

For all treatments, crop residues were not removed or grazed and negligible K losses from leaching and/or runoff were assumed. Annual budgets were estimated from crop grain yield and grain K concentrations data. No differences in grain K content between Control and NPS treatments were observed during the first years (2000–2002), and no grain-K measurements were made for the 2003–2009 period. For grain crops, provided crops evidence of K sufficiency, seed K contents are highly conserved that other management practices impacting yields are often inconsequential for grain K concentration^46–49^. Therefore, average values (2000–2002) of 4.0, 5.7 and 19.0 g K kg^−1^ grain for maize, wheat and soybean, respectively, were assumed to be constant for K removal calculations. Those values are in range with previously published data for maize^2^, wheat^3^, and soybean^4,50^.

Soil samples were collected at early spring in 2000, 2004 (0–20 cm) and 2009 (0–100 cm, see below) for all treatments. Soil properties related to total organic C, soil-test P^51^, soil pH^52^ (1:2.5 soil:water), bulk density^53^, and secondary elements^54,55^ were determined at the onset of the trial (2000). Soil extractable-K was determined by the pH 7 buffered 1.0 M ammonium-acetate method (NH_4_-OAc-K)^56^. Soil texture was measured also at the onset of the trial at topsoil (0–20 cm) and the rest of the soil layers (20–100 cm) were obtained from the Soil Survey Summary of the National Institute of Agricultural Technology (Instituto Nacional de Tecnología Agropecuaria, INTA).

In 2009, three sub-samples of soil profiles per plot were sampled and fresh mixed at five depths (0–20, 20–40, 40–60, 60–80, and 80–100 cm), dried at 40 °C until constant weight, and sieved (2 mm). Soil extractable-K was determined as NH_4_-OAc-K^50^, and by the modified sodium tetraphenyl boron method (NaBPh_4_-K)^9^. The vertical distribution of both NH_4_-OAc-K and NaBPh_4_-K was expressed on a volumetric basis (g m^−2^) as in Jobbagy and Jackson^16^. Bulk density, determined for Control and NPS plots at the onset of the study for each soil layer (Table 1), was used to transform from gravimetric (mg kg^−1^) to volumetric units (g m^−2^).

### Statistical analysis

For testing the first hypothesis, variables of interest were the topsoil NH_4_OAc-K (0–20 cm) for 2000, 2004 and 2009 years and the apparent cumulative K budget (kg ha^−1^) of both the Control and NPS treatments. For the apparent cumulative K budget, treatment with two levels (Control and NPS), and the crop number with 12 (La Blanca, La Hansa) and 13 (Balducchi and San Alfredo) levels (2000–2008) and their interactions were considered the fixed factors while the block was considered as random. For topsoil NH_4_OAc-K, treatment with two levels (Control and NPS), year with three levels (2000, 2004 and 2009) and their interactions were considered as fixed, while block was considered as random. In both cases, a mixed effect model with repeated measures over time was applied using the *nlme* package^56^ of the R software^57^. Additionally, the relationship between the NH_4_-OAc-K with the apparent cumulative K budgets (2000, 2004 and 2009) was tested with a simple linear regression model by pooling data from all four locations.

For the second hypothesis, NH_4_-OAc-K and NaBPh_4_-K contents (g m^−2^) were considered to evaluate the agricultural footprint (Pristine, Control and NPS in 2009). The analysis of variance (ANOVA) was partitioned by location. For vertical distributions of NH_4_-OAc-K and NaBPh_4_-K, we applied a mixed effect models with repeated measures over space (soil depth) using the *nlme* package^56^. Thereby, treatment, soil depth and their interactions were considered as fixed factors, while block was considered as random factor. The fulfillment of the assumptions of normality and homogeneity of variance for the K budget, NH_4_-OAc-K and NaBPh_4_-K were visually (QQ-plots and Student’s residuals vs. fitted values) and formally evaluated (Shapiro-Wilks normality test). Finally, we selected the best models based on the lowest scores for the Akaike Information Criterion (AIC). Means and significant interactions were obtained using the LSMEAN/PDIFF procedure using the *emmeans* package^58^.

For the third hypothesis, regression analyses were executed to estimate the effect of soil texture (clay, silt and sand -%-) and pristine K richness (Pristine NH_4_-OAc-K and NaBPh_4_-K) on the depletion of NH_4_-OAc-K and NaBPh_4_-K. A stepwise selection was applied using the *stepAIC* function from the MASS package^59^ to define the most relevant variables linearly associated with both depletions. Once variables were selected, partial linear and linear-plateau relationships were further tested for interpretation purposes using the *stats* package v3.6.2^57^.

## Supplementary Information

Supplementary Information 1.
