# Supplementary material for: Subsoil-potassium depletion accounts for the nutrient budget in high-potassium agricultural soils
Source: Sci Rep. 2021 Jun 2;11:11597. doi: 10.1038/s41598-021-90297-1 (PMC8172836; doi:10.1038/s41598-021-90297-1)
Supplement: Supplementary file 1 — Supplementary Information 1. [file 41598_2021_90297_MOESM1_ESM.docx]

**Supplementary Material**

**Subsoil-potassium depletion accounts for the nutrient budget in high-potassium agricultural soils**

**Adrián A. Correndo^1,*^, Gerardo Rubio^2^, Fernando O. García^3^ and Ignacio A. Ciampitti^1.*^**

**Correnponding:** [**correndo@ksu.edu**](mailto:correndo@ksu.edu) **–** [**ciampitti@ksu.edu**](mailto:ciampitti@ksu.edu) **– rubio@agro.uba.ar**

**Appendix A. ANOVAS**

A. Cumulative K budget (CBK)(Figure 1)……………………………………………………………………2

B. Topsoil NH4OAc-K (Figure 1)…………………………………………………………………………………………3

C. NH4OAc-K stocks 0-100 cm (Table 2)………………………………………………………………………4

D. NH4OAc-K stocks 0-20 vs 20-100 cm (Table 2)………………………………………………5

E. NaBPh4-K stocks 0-100 cm (Table 2)………………………………………………………………………6

F. NaBPh4-K stocks 0-20 vs. 0-100 cm (Table 2)………………………………………………7

G. NH4OAc-K vertical distribution(Figure 2)………………………………………………………8

H. NaBPh4-K vertical distribution (Figure 3)……………………………………………………9

**A. Cumulative K budget (CBK)(Figure 1)**

[1] "Balducchi NH4 CBKsoil"

Analysis of Deviance Table (Type III tests)

Response: CBK

Chisq Df Pr(>Chisq)

(Intercept) 0.00 1 1

TREAT 0.00 1 1

as.factor(SEQ) 457.64 2 <2e-16 ***

TREAT:as.factor(SEQ) 219.99 2 <2e-16 ***

---

Signif. codes: 0 ‘***’ 0.001 ‘**’ 0.01 ‘*’ 0.05 ‘.’ 0.1 ‘ ’ 1

[2] "San Alfredo NH4 CBKsoil"

Analysis of Deviance Table (Type III tests)

Response: CBK

Chisq Df Pr(>Chisq)

(Intercept) 0.00 1 1

TREAT 0.00 1 1

as.factor(SEQ) 6442.15 2 <2e-16 ***

TREAT:as.factor(SEQ) 524.21 2 <2e-16 ***

---

Signif. codes: 0 ‘***’ 0.001 ‘**’ 0.01 ‘*’ 0.05 ‘.’ 0.1 ‘ ’ 1

[3] "La Blanca NH4 CBKsoil"

Analysis of Deviance Table (Type III tests)

Response: CBK

Chisq Df Pr(>Chisq)

(Intercept) 0.00 1 1

TREAT 0.00 1 1

as.factor(SEQ) 681.63 2 < 2.2e-16 ***

TREAT:as.factor(SEQ) 36.34 2 1.285e-08 ***

---

Signif. codes: 0 ‘***’ 0.001 ‘**’ 0.01 ‘*’ 0.05 ‘.’ 0.1 ‘ ’ 1

[4] "La Hansa NH4 CBKsoil"

Analysis of Deviance Table (Type III tests)

Response: CBK

Chisq Df Pr(>Chisq)

(Intercept) 0.00 1 1

TREAT 0.00 1 1

as.factor(SEQ) 3961.94 2 <2e-16 ***

TREAT:as.factor(SEQ) 343.62 2 <2e-16 ***

---

Signif. codes: 0 ‘***’ 0.001 ‘**’ 0.01 ‘*’ 0.05 ‘.’ 0.1 ‘ ’ 1

**B. Topsoil NH4OAc-K (Figure 1)**

[1] "Balducchi NH4 topsoil"

Analysis of Deviance Table (Type III tests)

Response: NH4

Chisq Df Pr(>Chisq)

(Intercept) 452.0887 1 < 2.2e-16 ***

TREAT 0.0000 1 1.000000

as.factor(SEQ) 11.3843 2 0.003372 **

TREAT:as.factor(SEQ) 8.1215 2 0.017236 *

---

Signif. codes: 0 ‘***’ 0.001 ‘**’ 0.01 ‘*’ 0.05 ‘.’ 0.1 ‘ ’ 1

[2] "San Alfredo NH4 topsoil"

Analysis of Deviance Table (Type III tests)

Response: NH4

Chisq Df Pr(>Chisq)

(Intercept) 487.7787 1 < 2.2e-16 ***

TREAT 0.0000 1 1.000000

as.factor(SEQ) 10.2858 2 0.005841 **

TREAT:as.factor(SEQ) 2.4649 2 0.291575

---

Signif. codes: 0 ‘***’ 0.001 ‘**’ 0.01 ‘*’ 0.05 ‘.’ 0.1 ‘ ’ 1

[3] "La Blanca NH4 topsoil"

Analysis of Deviance Table (Type III tests)

Response: NH4

Chisq Df Pr(>Chisq)

(Intercept) 720.8825 1 <2e-16 ***

TREAT 0.0000 1 1.0000

as.factor(SEQ) 1.5978 2 0.4498

TREAT:as.factor(SEQ) 1.0066 2 0.6045

---

Signif. codes: 0 ‘***’ 0.001 ‘**’ 0.01 ‘*’ 0.05 ‘.’ 0.1 ‘ ’ 1

[4] "La Hansa NH4 topsoil"

Analysis of Deviance Table (Type III tests)

Response: NH4

Chisq Df Pr(>Chisq)

(Intercept) 1168.2835 1 <2e-16 ***

TREAT 0.0000 1 1.0000

as.factor(SEQ) 117.7458 2 <2e-16 ***

TREAT:as.factor(SEQ) 0.7943 2 0.6722

---

Signif. codes: 0 ‘***’ 0.001 ‘**’ 0.01 ‘*’ 0.05 ‘.’ 0.1 ‘ ’ 1

**C. NH4OAc-K stocks 0-100 cm (Table 2)**

[1] "ANOVA Balducchi"

Analysis of Deviance Table (Type III tests)

Response: NH4OAc

Chisq Df Pr(>Chisq)

(Intercept) 56.401 1 5.909e-14 ***

TREAT 111.568 2 < 2.2e-16 ***

---

Signif. codes: 0 ‘***’ 0.001 ‘**’ 0.01 ‘*’ 0.05 ‘.’ 0.1 ‘ ’ 1

[2] "ANOVA San Alfredo"

Analysis of Deviance Table (Type III tests)

Response: NH4OAc

Chisq Df Pr(>Chisq)

(Intercept) 2230.01 1 < 2.2e-16 ***

TREAT 161.65 2 < 2.2e-16 ***

---

Signif. codes: 0 ‘***’ 0.001 ‘**’ 0.01 ‘*’ 0.05 ‘.’ 0.1 ‘ ’ 1

[3] "ANOVA La Blanca"

Analysis of Deviance Table (Type III tests)

Response: NH4OAc

Chisq Df Pr(>Chisq)

(Intercept) 352.403 1 < 2.2e-16 ***

TREAT 32.842 2 7.386e-08 ***

---

Signif. codes: 0 ‘***’ 0.001 ‘**’ 0.01 ‘*’ 0.05 ‘.’ 0.1 ‘ ’ 1

[4] "ANOVA La Hansa"

Analysis of Deviance Table (Type III tests)

Response: NH4OAc

Chisq Df Pr(>Chisq)

(Intercept) 379.556 1 < 2.2e-16 ***

TREAT 82.993 2 < 2.2e-16 ***

---

Signif. codes: 0 ‘***’ 0.001 ‘**’ 0.01 ‘*’ 0.05 ‘.’ 0.1 ‘ ’ 1

**D. NH4OAc-K stocks 0-20 vs 20-100 cm (Table 2)**

[1] "ANOVA Balducchi"

Analysis of Deviance Table (Type III tests)

Response: NH4OAc

Chisq Df Pr(>Chisq)

(Intercept) 122.5607 1 < 2.2e-16 ***

TREAT 6.2049 2 0.04494 *

as.factor(DEPTH) 453.0962 1 < 2.2e-16 ***

TREAT:as.factor(DEPTH) 23.3782 2 8.385e-06 ***

---

Signif. codes: 0 ‘***’ 0.001 ‘**’ 0.01 ‘*’ 0.05 ‘.’ 0.1 ‘ ’ 1

[2] "ANOVA San Alfredo"

Analysis of Deviance Table (Type III tests)

Response: NH4OAc

Chisq Df Pr(>Chisq)

(Intercept) 265.0964 1 <2e-16 ***

TREAT 78.8217 2 <2e-16 ***

as.factor(DEPTH) 876.2072 1 <2e-16 ***

TREAT:as.factor(DEPTH) 3.2993 2 0.1921

---

Signif. codes: 0 ‘***’ 0.001 ‘**’ 0.01 ‘*’ 0.05 ‘.’ 0.1 ‘ ’ 1

[3] "ANOVA La Blanca"

Analysis of Deviance Table (Type III tests)

Response: NH4OAc

Chisq Df Pr(>Chisq)

(Intercept) 398.6729 1 < 2.2e-16 ***

TREAT 2.8963 2 0.235

as.factor(DEPTH) 1070.5360 1 < 2.2e-16 ***

TREAT:as.factor(DEPTH) 21.9442 2 1.717e-05 ***

---

Signif. codes: 0 ‘***’ 0.001 ‘**’ 0.01 ‘*’ 0.05 ‘.’ 0.1 ‘ ’ 1

[4] "ANOVA La Hansa"

Analysis of Deviance Table (Type III tests)

Response: NH4OAc

Chisq Df Pr(>Chisq)

(Intercept) 33.3053 1 7.877e-09 ***

TREAT 5.7746 2 0.05573 .

as.factor(DEPTH) 577.3584 1 < 2.2e-16 ***

TREAT:as.factor(DEPTH) 3.1495 2 0.20706

---

Signif. codes: 0 ‘***’ 0.001 ‘**’ 0.01 ‘*’ 0.05 ‘.’ 0.1 ‘ ’ 1

**E. NaBPh4-K stocks 0-100 cm (Table 2)**

[1] "ANOVA Balducchi"

Analysis of Deviance Table (Type III tests)

Response: NaBPh4

Chisq Df Pr(>Chisq)

(Intercept) 2798.15 1 < 2.2e-16 ***

TREAT 850.71 2 < 2.2e-16 ***

---

Signif. codes: 0 ‘***’ 0.001 ‘**’ 0.01 ‘*’ 0.05 ‘.’ 0.1 ‘ ’ 1

[2] "ANOVA San Alfredo"

Analysis of Deviance Table (Type III tests)

Response: NaBPh4

Chisq Df Pr(>Chisq)

(Intercept) 3689.1565 1 < 2e-16 ***

TREAT 9.6218 2 0.00814 **

---

Signif. codes: 0 ‘***’ 0.001 ‘**’ 0.01 ‘*’ 0.05 ‘.’ 0.1 ‘ ’ 1

[3] "ANOVA La Blanca"

Analysis of Deviance Table (Type III tests)

Response: NaBPh4

Chisq Df Pr(>Chisq)

(Intercept) 292.6722 1 <2e-16 ***

TREAT 8.8125 2 0.0122 *

---

Signif. codes: 0 ‘***’ 0.001 ‘**’ 0.01 ‘*’ 0.05 ‘.’ 0.1 ‘ ’ 1

[4] "ANOVA La Hansa"

Analysis of Deviance Table (Type III tests)

Response: NaBPh4

Chisq Df Pr(>Chisq)

(Intercept) 3284.97 1 < 2.2e-16 ***

TREAT 119.73 2 < 2.2e-16 ***

---

Signif. codes: 0 ‘***’ 0.001 ‘**’ 0.01 ‘*’ 0.05 ‘.’ 0.1 ‘ ’ 1

**F. NaBPh4-K stocks 0-20 vs. 0-100 cm (Table 2)**

[1] "ANOVA Balducchi"

Analysis of Deviance Table (Type III tests)

Response: NaBPh4

Chisq Df Pr(>Chisq)

(Intercept) 451.5350 1 < 2e-16 ***

TREAT 7.5416 2 0.02303 *

as.factor(DEPTH) 380.0700 1 < 2e-16 ***

TREAT:as.factor(DEPTH) 551.3750 2 < 2e-16 ***

---

Signif. codes: 0 ‘***’ 0.001 ‘**’ 0.01 ‘*’ 0.05 ‘.’ 0.1 ‘ ’ 1

[2] "ANOVA San Alfredo"

Analysis of Deviance Table (Type III tests)

Response: NaBPh4

Chisq Df Pr(>Chisq)

(Intercept) 397.5766 1 < 2e-16 ***

TREAT 0.5656 2 0.75367

as.factor(DEPTH) 1443.0417 1 < 2e-16 ***

TREAT:as.factor(DEPTH) 5.7321 2 0.05692 .

---

Signif. codes: 0 ‘***’ 0.001 ‘**’ 0.01 ‘*’ 0.05 ‘.’ 0.1 ‘ ’ 1

[3] "ANOVA La Blanca"

Analysis of Deviance Table (Type III tests)

Response: NaBPh4

Chisq Df Pr(>Chisq)

(Intercept) 253.0164 1 < 2.2e-16 ***

TREAT 9.3436 2 0.009356 **

as.factor(DEPTH) 1158.9464 1 < 2.2e-16 ***

TREAT:as.factor(DEPTH) 46.6989 2 7.235e-11 ***

---

Signif. codes: 0 ‘***’ 0.001 ‘**’ 0.01 ‘*’ 0.05 ‘.’ 0.1 ‘ ’ 1

[4] "ANOVA La Hansa"

Analysis of Deviance Table (Type III tests)

Response: NaBPh4

Chisq Df Pr(>Chisq)

(Intercept) 16.9654 1 3.807e-05 ***

TREAT 1.8517 2 0.39619

as.factor(DEPTH) 100.2641 1 < 2.2e-16 ***

TREAT:as.factor(DEPTH) 6.4561 2 0.03963 *

---

Signif. codes: 0 ‘***’ 0.001 ‘**’ 0.01 ‘*’ 0.05 ‘.’ 0.1 ‘ ’ 1

**G. NH4OAc-K vertical distribution(Figure 2)**

[1] "ANOVA Balducchi"

Analysis of Deviance Table (Type III tests)

Response: NH4OAc

Chisq Df Pr(>Chisq)

(Intercept) 102.395 1 < 2.2e-16 ***

TREAT 39.994 2 2.067e-09 ***

as.factor(DEPTH) 20.460 4 0.0004052 ***

TREAT:as.factor(DEPTH) 21.827 8 0.0052473 **

---

Signif. codes: 0 ‘***’ 0.001 ‘**’ 0.01 ‘*’ 0.05 ‘.’ 0.1 ‘ ’ 1

[2] "ANOVA San Alfredo"

Analysis of Deviance Table (Type III tests)

Response: NH4OAc

Chisq Df Pr(>Chisq)

(Intercept) 3521.78 1 < 2.2e-16 ***

TREAT 315.45 2 < 2.2e-16 ***

as.factor(DEPTH) 368.66 4 < 2.2e-16 ***

TREAT:as.factor(DEPTH) 146.31 8 < 2.2e-16 ***

---

Signif. codes: 0 ‘***’ 0.001 ‘**’ 0.01 ‘*’ 0.05 ‘.’ 0.1 ‘ ’ 1

[3] "ANOVA La Blanca"

Analysis of Deviance Table (Type III tests)

Response: NH4OAc

Chisq Df Pr(>Chisq)

(Intercept) 241.848 1 < 2.2e-16 ***

TREAT 21.394 2 2.261e-05 ***

as.factor(DEPTH) 14.594 4 0.005621 **

TREAT:as.factor(DEPTH) 52.957 8 1.099e-08 ***

---

Signif. codes: 0 ‘***’ 0.001 ‘**’ 0.01 ‘*’ 0.05 ‘.’ 0.1 ‘ ’ 1

[4] "ANOVA La Hansa"

Analysis of Deviance Table (Type III tests)

Response: NH4OAc

Chisq Df Pr(>Chisq)

(Intercept) 309.166 1 < 2.2e-16 ***

TREAT 50.791 2 9.353e-12 ***

as.factor(DEPTH) 16.109 4 0.002876 **

TREAT:as.factor(DEPTH) 21.773 8 0.005355 **

---

Signif. codes: 0 ‘***’ 0.001 ‘**’ 0.01 ‘*’ 0.05 ‘.’ 0.1 ‘ ’ 1

**H. NaBPh4-K vertical distribution (Figure 3)**

[1] "ANOVA Balducchi"

Analysis of Deviance Table (Type III tests)

Response: NaBPh4-K

Chisq Df Pr(>Chisq)

(Intercept) 473.9849 1 < 2.2e-16 ***

TREAT 8.8719 2 0.0118439 *

as.factor(DEPTH) 23.0697 4 0.0001226 ***

TREAT:as.factor(DEPTH) 72.9667 8 1.258e-12 ***

---

Signif. codes: 0 ‘***’ 0.001 ‘**’ 0.01 ‘*’ 0.05 ‘.’ 0.1 ‘ ’ 1

[2] "ANOVA San Alfredo"

Analysis of Deviance Table (Type III tests)

Response: NaBPh4-K

Chisq Df Pr(>Chisq)

(Intercept) 567.7383 1 <2e-16 ***

TREAT 0.5123 2 0.7740

as.factor(DEPTH) 7.4895 4 0.1122

TREAT:as.factor(DEPTH) 2.6759 8 0.9530

---

Signif. codes: 0 ‘***’ 0.001 ‘**’ 0.01 ‘*’ 0.05 ‘.’ 0.1 ‘ ’ 1

[3] "ANOVA La Blanca"

Analysis of Deviance Table (Type III tests)

Response: NaBPh4-K

Chisq Df Pr(>Chisq)

(Intercept) 284.8824 1 < 2.2e-16 ***

TREAT 10.9012 2 0.0042938 **

as.factor(DEPTH) 9.1911 4 0.0564972 .

TREAT:as.factor(DEPTH) 31.1049 8 0.0001346 ***

---

Signif. codes: 0 ‘***’ 0.001 ‘**’ 0.01 ‘*’ 0.05 ‘.’ 0.1 ‘ ’ 1

[4] "ANOVA La Hansa"

Analysis of Deviance Table (Type III tests)

Response: NaBPh4-K

Chisq Df Pr(>Chisq)

(Intercept) 502.8823 1 < 2.2e-16 ***

TREAT 5.9082 2 0.05213 .

as.factor(DEPTH) 34.0389 4 7.316e-07 ***

TREAT:as.factor(DEPTH) 7.2642 8 0.50841

---

Signif. codes: 0 ‘***’ 0.001 ‘**’ 0.01 ‘*’ 0.05 ‘.’ 0.1 ‘ ’ 1
